# *Trichosporon asahii* causes scalp infections in a child with atopic dermatitis: A case report

**DOI:** 10.1016/j.idcr.2025.e02358

**Published:** 2025-09-08

**Authors:** Panling Wei, Xiaoli Yang, Zaixing Wang

**Affiliations:** a Department of Dermatology, The First Affiliated Hospital of Anhui Medical University, Hefei, Anhui, China; b Institute of Dermatology, Anhui Medical University, Hefei, Anhui, China; c Key Laboratory of Dermatology (Anhui Medical University), Ministry of Education, Hefei, Anhui, China

**Keywords:** *Trichosporon asahii*, Child, Scalp infection, Atopic dermatitis, Itraconazole

## Abstract

This report describes a 4-year-old boy with a history of atopic dermatitis who developed a scalp infection caused by *Trichosporon asahii*. The patient presented with scalp lesions and fever. Empirical antibacterial and antifungal therapy failed to improve his symptoms, prompting further investigation. Culture of scalp lesion exudate grew *Trichosporon asahii*. The patient received combination therapy and achieved clinical improvement. This case highlights the diagnostic and therapeutic challenges posed by rare fungal infections. Early recognition and timely intervention are essential to achieving optimal outcomes in such complex cases.

## Introduction

*Trichosporon asahii* is a rare and emerging yeast-like fungus of the genus Trichosporon, order Trichosporonales, class Tremellomycetes (phylum Basidiomycota) that is widely found in water, soil, and vegetation and is known to colonize the skin and respiratory, genitourinary, and gastrointestinal tracts [1]. In recent years, the incidence of *Trichosporon asahii* infection has been increasing year by year, typically affecting immunocompromised patients [2]. *Trichosporon asahii* has been reported to cause potentially fatal disseminated infections affecting the brain, heart, lungs, liver, kidneys, and skin [3]. Despite the fact that *Trichosporon asahii* is more common in deeply invasive infections and can also cause superficial infections in healthy people, it is extremely rare. The most common superficial infection is white piedra, which is characterized by white to light brown nodules loosely attached to the hair shaft. It can disseminate in neutropenic or HIV-infected individuals and manifests as extensive papular or purpuric nodules [4]. This article describes a case of scalp infection caused by *Trichosporon asahii* infection in a child with atopic dermatitis, highlighting the challenges of diagnosis and treatment.

## Case

**Fig. 1:**
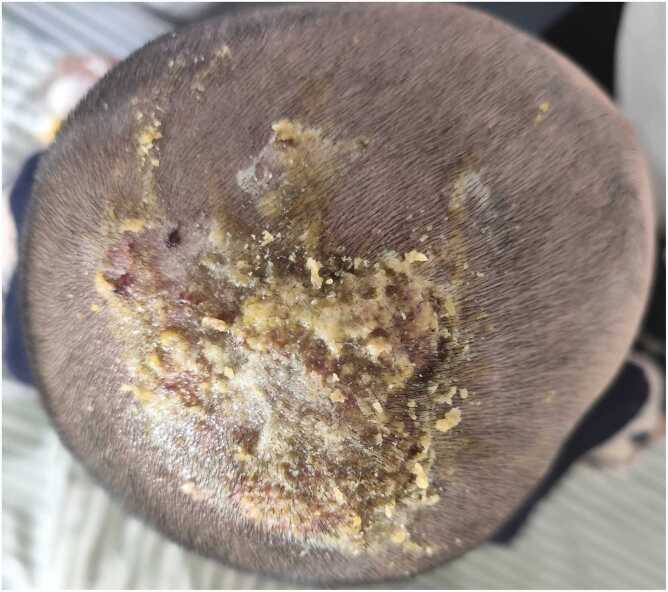
Day 1 of hospitalization: multiple inflammatory nodules on the top of the head, superficial erosions and purulent discharge.

A 4-year-old boy with a history of atopic dermatitis was admitted with a chief complaint of “painful scalp lesions for 2 weeks and scattered pruritic rash on the trunk and extremities for 3 days.” Fourteen days before admission (Day −14), his parents noticed patchy dark-red papules and plaques on the vertex of the scalp, which gradually enlarged and ulcerated, accompanied by yellowish and bloody exudation and marked local tenderness. Enlargement of the postauricular and occipital superficial lymph nodes was also noted. The patient had been treated with oral cefaclor and topical mupirocin ointment, sulfur ointment, and butyrate hydrocortisone cream, but the lesions did not improve. Moreover, diffuse pale-red papules gradually appeared over the body. On the day of admission (Day 0), the patient’s vital signs were as follows: temperature 37.5 °C, pulse 122 beats per minute, respiratory rate 22 breaths per minute, and blood pressure 85/58 mmHg. His height was 110 cm and weight was 20 kg. A complete blood count performed at the same time (Day 0) revealed leukocytosis (14.22 × 10⁹/L; reference range, 4.4–11.9 × 10⁹/L) with elevated neutrophil percentage (75.1 %; reference range, 22–65 %). Direct potassium hydroxide (KOH) examination of scalp scales demonstrated positive hyphae but no spores, and no fungal structures were observed in broken hairs. A preliminary diagnosis of “kerion and skin infection” was made, and the patient was admitted to our department for further management. On physical examination, multiple inflammatory nodules with erosion and purulent exudation were observed on the vertex of the scalp (Fig. 1). Scattered pale-red papules were present on the trunk and extremities, and enlargement of the postauricular and occipital lymph nodes was evident; a chest X-ray performed after admission showed no significant pulmonary abnormalities, and the remainder of the systemic examination was unremarkable.

**Fig. 2:**
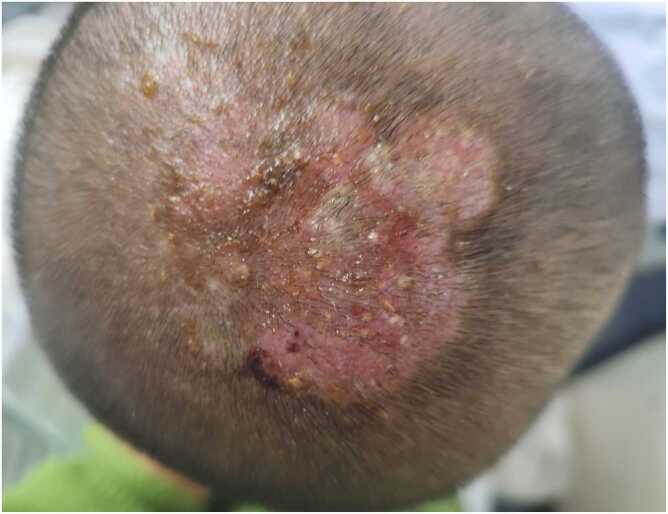
On the 5th day of hospitalization, inflammatory nodules based on flaky erythema on the top of the head, decreased purulent discharge, and diffuse alopecia.

Initial treatment consisted of oral terbinafine, topical chlortetracycline eye ointment, topical miconazole nitrate cream, and empiric intravenous ceftriaxone. On Day +5, the patient developed fever. Physical examination revealed inflammatory nodules on erythematous plaques at the vertex, decreased purulent exudation, and diffuse alopecia (Fig. 2). Red papules on the trunk and face became more prominent. Blood cultures (four bottles: two aerobic and two anaerobic bottles from bilateral arms), influenza A antigen, and SARS-CoV-2 nucleic acid tests were all negative. Repeated cultures of scalp exudate grew *Trichosporon asahii*, identified by matrix-assisted laser desorption/ionization time-of-flight mass spectrometry (MALDI-TOF MS). Antifungal susceptibility testing was performed using the broth microdilution method according to CLSI guidelines. Due to the absence of official clinical breakpoints for *Trichosporon asahii*, epidemiological cutoff values (ECVs) were applied for interpretation. Minimum inhibitory concentrations (MICs) for antifungal drugs were as follows: amphotericin B, 0.12 µg/mL; fluconazole, 0.12 µg/mL; voriconazole, 0.015 µg/mL; itraconazole, 0.03 µg/mL. Repeat complete blood count revealed leukocytosis (13.28 × 10⁹/L) with neutrophilia (73.1 %). Serum IgE was elevated at 190.82 IU/mL.

**Fig. 3:**
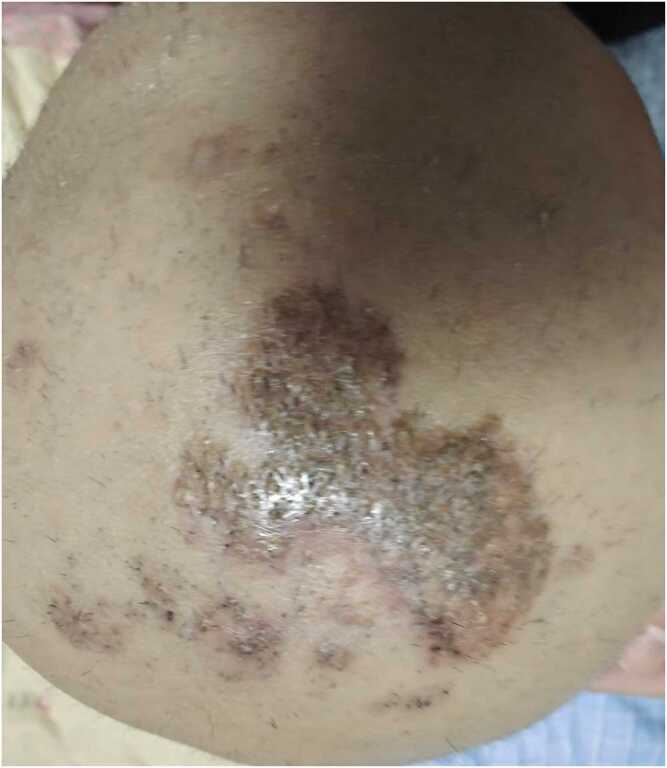
After 2 months of systematic treatment, dark erythema on the top of the head, yellow crusts attached to the surface, and sparse new hairs were visible in the diffuse alopecia.

Treatment was adjusted to oral itraconazole (50 mg twice daily) and subcutaneous dupilumab (300 mg) to control atopic inflammation, along with oral cetirizine as an antihistamine. The patient continued to have intermittent fever. Further investigations, including immunoglobulin levels, complement, lymphocyte subsets, and autoantibody tests, showed no abnormalities. He was referred to the National Clinical Research Center for Skin and Immune Diseases (Chinese Academy of Medical Sciences, Peking Union Medical College) for further treatment. Two days after transfer (Day +7), his temperature returned to normal, the scattered pale-red papules on the trunk and extremities had significantly improved，with near-complete resolution observed by the second week of therapy (Day +14). Oral itraconazole was continued (0.1 g daily), combined with oral prednisone (10 mg daily) to control inflammation. The scalp was shaved, and topical bifonazole cream was applied. At 2-month follow-up (Day +60), examination revealed pale-red plaques with yellowish crusts on the vertex, with new hair growth in previously alopecic areas (Fig. 3). Repeated KOH examinations during follow-up were negative. Prednisone was gradually tapered to maintenance doses, and dupilumab therapy was continued to control atopic dermatitis. The patient’s condition remained stable.

## Discussion

In this case, a child with atopic dermatitis was infected with *Trichosporon asahii*, and developed scalp inflammatory nodules, erosions, exudations, and lymphadenopathy-like manifestations of tinea capitis, and did not respond well to conventional local antifungal therapy, which included oral terbinafine, topical miconazole nitrate cream. Traditionally, non-dermatophytes (e.g. yeasts or other opportunistic pathogens) have been thought to be extremely rare in superficial scalp infections, but in recent years, with the change in the spectrum of fungal infections, the increase in antibiotic abuse, and immune-related diseases, some atypical fungi have been shown to cause skin and even hair infections [5].

*Trichosporon asahii* is a yeast-like fungus of the order Trichosporonales, which is widely found in nature, including water, soil, air, and normal flora of human skin and gastrointestinal tract [6]. In the immunosuppressed state, *Trichosporon asahii* often acts as an opportunistic pathogen to cause severe or even fatal disseminated infection, affecting multiple organs such as the lungs, liver, kidneys, and central nervous system, with a high mortality rate [7]. Although rare, superficial infections in immunocompetent individuals have been reported, typically involving mucocutaneous sites [8] or medical devices (urinary catheters [9] and central venous catheters [10**]**), and are often misdiagnosed.

Treatment of trichosporonosis is still challenging, with antifungal therapy as the mainstay. Superficial infections are treated with topical antifungal drugs, but hair infections may recur after topical therapy alone, so it has been suggested that hair removal and systemic antifungal therapy should be used in conjunction with shaving [11]. A growing body of data suggests that amphotericin B has a very limited therapeutic effect on *Trichosporon spp*., including *Trichosporon asahii*, and that triazoles have better in vitro and in vivo antifungal activity than amphotericin B [12], and in vitro studies have shown that voriconazole exhibits optimal antifungal activity against different *Trichosporon spp*. compared with amphotericin B and fluconazole [13]. It is worth noting that newer triazole agents, such as isavuconazole, have also demonstrated promising in vitro activity against *Trichosporon spp*., with generally low minimum inhibitory concentration (MIC) values, suggesting their potential as alternative treatment options [14]. These findings indicate that voriconazole and some of the newer azole agents may hold significant potential in the treatment of related infections, providing further support for their clinical application. In this case, initial treatment with topical therapy was ineffective. Based on pathogen identification by MALDI-TOF MS and antifungal susceptibility testing (itraconazole MIC = 0.03 μg/mL), systemic itraconazole therapy was promptly initiated. This regimen, combined with oral prednisone and dupilumab to synergistically control allergic and inflammatory responses, led to rapid clinical improvement. This case highlights that in refractory tinea capitis, the possibility of non-dermatophyte infections should be considered, and precise pathogen identification along with susceptibility results are critical for guiding clinical therapeutic decisions.

In addition, the child's previous history of allergic diseases such as atopic dermatitis and allergic rhinitis suggests that the skin barrier function may be mildly impaired, and may also create an opportunity for the pathogenesis of *Trichosporon asahii*. This observation is consistent with reports that even immunocompetent people can develop Trichosporon infections in the setting of impaired skin integrity and local environmental changes [15].

## Conclusion

This case suggests that clinicians should be alert to the possibility of atypical pathogenic fungi for patients with refractory tinea capitis who do not respond to conventional treatment. Early fungal microscopy and culture, identification of pathogen types, and reasonable selection of antifungal drugs and adjuvant anti-inflammatory therapy can help improve the success rate of treatment and reduce the risk of complications and misdiagnosis and mistreatment.

## CRediT authorship contribution statement

**Zaixing Wang:** Writing – review & editing, Supervision. **Xiaoli Yang:** Writing – review & editing. **Panling Wei:** Writing – original draft.

## Author statement

Panling Wei and Xiaoli Yang are responsible for manuscript integrity and contributed equally to diagnosis, treatment, analysis, and writing. Wang Z supervised the clinical management and reviewed the manuscript. All authors approved the final version, confirm originality and no conflict of interest, and consent to submission. Patient consent was obtained and data are available on request.

## Ethical approval

Not applicable. Written informed consent was obtained from the patient for publication of this case report and accompanying images.

## Consent

Informed consent was obtained from the patient for publication of this case report and any accompanying images.

## Funding

No funding was provided.

## Declaration of Competing Interest

The authors have no conflicts of interest to declare.
